# The Effects of the Compassionate Mind Training for Caregivers on Professional Quality of Life and Mental Health: Outcomes from a Cluster Randomized Trial in Residential Youth Care Settings

**DOI:** 10.1007/s10566-023-09749-6

**Published:** 2023-05-03

**Authors:** Laura Santos, Maria do Rosário Pinheiro, Daniel Rijo

**Affiliations:** Center for Research in Neuropsychology and Cognitive and Behavioral Intervention, Faculty of Psychology and Educational Sciences, University of Coimbra, Rua do Colégio Novo, 3030-115 Coimbra, Portugal

**Keywords:** Residential youth care, Caregivers, Compassion, Mental health, Professional quality of life, Compassionate mind training

## Abstract

**Background:**

Psychological distress is highly noticeable among caregivers working in residential youth care (RYC). Maintaining and enhancing caregivers’ professional mental health and quality of life is crucial to achieve effective outcomes in RYC. Nevertheless, trainings to protect caregivers’ mental health are scarce. Considering the buffering effect over negative psychological outcomes, compassion training could be beneficial in RYC.

**Objective:**

This study is part of a Cluster Randomized Trial examining the effects of the Compassionate Mind Training for Caregivers (CMT-Care Homes), looking at professional quality of life and mental health of caregivers working in RYC.

**Method:**

The sample was composed of 127 professional caregivers from 12 Portuguese residential care homes (RCH). RCHs were randomly allocated at experimental (N = 6) and control group (N = 6). Participants were assessed at baseline, post-treatment, and 3 and 6-month follow-ups, answering to the Professional Quality of Life Scale and the Depression, Anxiety and Stress Scale. Program effects were tested using a two-factor mixed MANCOVA, with self-critical attitude and education degree as covariates.

**Results:**

MANCOVA showed a significant Time × Group interaction effects (F = 1.890, *p* = .014; ${\eta }_{p}^{2}$ = .050), with CMT-Care Homes participants presenting lower scores on burnout, anxiety, and depression at 3 and 6-months follow-ups, when compared with controls. Participants that received CMT-Care Homes considered the program useful to deal with pandemic threats and with youth during lockdowns.

**Conclusion:**

This study shows the benefits of the CMT-Care Homes in helping professional caregivers reducing burnout, anxiety and depression, and dealing with pandemic challenges in RYC.

*Trial registration*: This cluster randomized trial was registered at ClinicalTrials.gov (TRN: NCT04512092) on 6th August 2020.

## Introduction

Residential youth care (RYC) is an alternative care response to youth with prior history of maltreatment, aiming to offer them a chance to have a healthy development and shape their future (Quality4Children, 2007). Research demonstrates that the resilience of those who suffer trauma can be enhanced if they are connected to a caring and responsive caregiver (Larkin et al., 2012).

Caregivers have a key role within RYC settings (Li & Julian, 2012; Santos et al., 2023c). Nevertheless, they face many challenges linked to the target group and working conditions, that might threaten both the quality of care and the relationships established not only with children but also among co-workers (Colton & Roberts, 2007; McElvaney & Tatlow-Golden, 2016). Specifically, caregivers are required to actively listen to youth previous experiences and, at the same time, they are expected to soothe them, offer counselling, and care without being overwhelmed by their own emotional responses (Pfaff et al., 2017). Caregivers have to deal with highly traumatized youth, frequently presenting mental health difficulties, while having to cope with episodes of aggression and disruptive behaviors (McElvaney & Tatlow-Golden, 2016). At the same time, they are expected to meet the complex needs and ensure the well-being of children and youth under their care (Barford & Whelton, 2010; Steinlin et al., 2017). Additionaly, working conditions are often poor and the care system frequently does not offer appropiate responses considereing the youth intervention needs (McElvaney & Tatlow-Golden, 2016). On the one hand, the high number of youths under care usually contributes to long hours of work which, combined with shifts, set the base for excessive workloads. On the other hand, the opportunities offered for professional and financial rewards are usually scarce for professionals working within these settings (Colton & Roberts, 2007). The lack of support among colleagues and also by the organization management, as well as communication barriers between professionals, have also been reported as a matter of concern (Del Valle et al., 2007; McElvaney & Tatlow-Golden, 2016).

The repeated exposure to emotional pain, in multiple stressful care situations, may lead caregivers to feel frustration, helplessness, and powerlessness, as well as to perceive the job as resulting in greater distress than satisfaction (Colton & Roberts, 2007; McElvaney & Tatlow-Golden, 2016; Stamm, 2010). As a result, caregivers are prone to experience stress, burnout, secondary traumatic stress (STS), anxiety, and depression (Barford & Whelton, 2010; Bürgin et al., 2020; Del Valle et al., 2007; Hermon & Chahla, 2019; Raskin et al., 2015; Santos et al., 2022a; Steinlin et al., 2017). Such conditions may lead to diminished care, loss of interest in others, negative attitudes towards work, and reduced personal accomplishment (Maslach et al., 2001), limiting both the ability to establish empathic relationships and the emotional availability to care (Bride et al., 2007). Besides affecting the quality of care (Steinlin et al., 2017), such condition may also lead to turnover, which is a common problem within RYC (Colton & Roberts, 2007), with negative consequences to the consistency of interventions (Barford & Whelton, 2010).

In addition to the multiple abovementioned work stressors, during the Covid-19 pandemic, residential care homes (RCH) were required to quickly adapt to the public health measures (e.g., lockdowns, closures of schools) and social distancing (Carvalho et al., 2022). As “frontline workers”, while dealing with the risk of becoming infected, professional caregivers had to ensure the provision of care, maintain daily routines as much as possible, and reassure children and youth, who also could be experiencing some psychological disturbance (Ravens-Sieberer et al., 2020). Hence, professionals had to take care of a more stressed group, with even less resources (due to isolations, medical discharges; Whitt-Woosley et al., 2022). These challenges, combined with uncertainty, resulted in additional fears, worries, and stress (Carvalho et al., 2022).

Altogether, the above-mentioned demands reinforce the need to offer proper training to support caregivers in high-stress work environments, as it is the case of RYC (Barford & Whelton, 2010; McElvaney & Tatlow-Golden, 2016). Although some studies tried to overcome this need, some limitations still persist: (1) existing research showed that interventions didn’t had the expected impact (Donald, 2015; Silva & Gaspar, 2014; Vallejos et al., 2016); (2) when positive effects on stress and related syndromes were reached, those studies presented methodological limitations, such as small sample size, lack of randomization and/or absence of control group (Hidalgo et al., 2016; Schmid et al., 2020; Turner, 2017; van Gink et al., 2018); (3) programs were specifically tailored to babies homes (St. Petersburg–USA Orphanage Research Team, 2008). Some trauma-informed approaches also addressed staff mental health. Yet, they also showed some limitations linked to implementation and evaluation (Raymond, 2020).

Compassion-based interventions have been delivered in organizations (Andersson et al., 2022) and in different caring-focused settings, showing a potential to improve care quality, strengthen relationships with clients, protect against burnout, and increase professionals’ well-being (Boellinghaus et al., 2014; Delaney, 2018; Maratos et al., 2019; Matos et al., 2022a; Sansó et al., 2017). This kind of interventions aim to cultivate compassion towards the self and towards others. Compassion can be defined as a motivation to be responsive to one´s own and others suffering and to act in order to alleviate or prevent it (Gilbert, 2020). It may occur in three interactive flows, involving giving compassion to others, receiving compassion from others, and being self-compassionate (Gilbert, 2019). Each of these flows may reveal associated fears, blocks and/or resistances (Gilbert et al., 2011).

Within helping settings, cultivating compassion towards others may facilitate the ‘self-other’ distinction, in order not to absorb others’ suffering or negative emotions as ours own (Vachon, 2016). In other words, if caregivers respond to youth’s suffering with compassion, they will empathize with the suffering, but not identify themselves with it; thus, they will be able to better regulate their own negative affect (Singer & Klimecki, 2014). Hence, compassion can be seen as an emotion-regulation strategy that regulates negative affect (Preckel et al., 2018) and protects against stress (Matos et al., 2017), and mental health problems (Irons & Heriot-Maitland, 2020; Kirby et al., 2017).

Also, relevant to helping professionals is the fact that individuals who give care to others, but do not seek care from others, reveal some difficulties in self-compassion and self-reassurance (Hermanto & Zuroff, 2016). Professionals who lack self-compassion, and particularly those expressing a higher self-critical attitude, are more susceptible to burnout as a result of continuously caring for others, while ignoring their own emotional needs (Gracia-Gracia & Oliván-Blázquez, 2017). In addition, those who lack self-compassion are not only more self-critical, but also more critical and controlling towards others (Gilbert et al., 2011), which may compromise both the establishment of secure relationships as the provision of appropriate care. Self-compassion involves relating to oneself with care and concern when facing hardship or perceived inadequacy (Neff, 2003), and it has been linked to increased well-being (Barnard & Curry, 2011), and lower levels of psychopathology, including anxiety and depression (Ferrari et al., 2019; MacBeth & Gumley, 2012; Wilson et al., 2019). At a professional level, self-compassion can help in coping with uncertain and challenging conditions, resulting in increased job satisfaction and professional well-being, as well as less burnout, depression, anxiety, and stress (Andersson et al., 2022; Babenko et al., 2019). Considering that self-care has been associated with caregivers’ quality of life (Sansó et al., 2015) and higher self-confidence as a caregiver (Bratt et al., 2019), motivating these professionals and raising their awareness on the need to care for their own well-being seems essential.

This study is part of a Cluster Randomized Trial (CRT) aiming to test the effectiveness of the Compassionate Mind Training for Caregivers (CMT-Care Homes) working within RYC settings in several compassion related variables (Santos et al., 2022b, 2023b). It expands compassion-based approaches to RYC settings. It tests the effectiveness of a new training program specifically designed for RYC staff, based in a well-established therapeutic model and practices, resorting to a CRT in a real-world setting. In the current work, the effects of the CMT-Care Homes will be investigated on caregivers’ professional quality of life and mental health symptoms, also investigating whether observed changes after program completion are sustained across time. It responds to the gaps reported in recent systematic reviews about the scarcity of evidence-based and manualized programs to protect and enhance the mental health of RYC professionals, who work in a highly stressful and emotionally demanding setting (Santos et al., 2023a). Considering that caregivers are key agents of change in RYC, this program may contribute to improve the care practices and to reach better outcomes for children and youth placed in RYC, by providing support to those who have been given the responsibility to help these youth to heal and thrive.

The main research questions of this study are: (1) How does the CMT-Care Homes impacts Professional Quality of life and Mental Health symptoms among caregivers working in RYC settings? (2) Are the effects of CMT-Care Homes in these variables sustained across time? Considering that the pandemic situation started during the current CRT, secondary questions are: (1) Was the CMT-Care Homes useful to deal with the pandemic situation? (2) Was the CMT-Care Homes useful to deal with children and youth within RCHs during lockdowns? In accordance with previous research testing compassion-based approaches in helping settings (Matos et al., 2022a; Sansó et al., 2017), it is hypothesized that the CMT-Care Homes will produce significant improvements in burnout, STS, depression, anxiety, and stress, as well as an increase in compassion satisfaction, when comparing caregivers who received the training with those in the control group. In addition, it is expected that the effects of attending the CMT-Care Homes will be maintained at 3 and 6-month follow-ups (Ferrari et al., 2019; Irons & Heriot-Maitland, 2020; Matos et al., 2022a).

## Method

This cluster randomized trial followed the standards of Consort 2010 statement: Extension to cluster randomized trials (Campbell et al., 2012). The current study was approved by the Ethics Committee of the Faculty of Psychology and Educational Sciences from the University of Coimbra (CEDI22.03.2018) and its procedures were in accordance with the Code of Ethics of the World Medical Association (Declaration of Helsinki) for experiments involving humans. The authors have no competing interests to declare that are relevant to the content of this article.

### Participants

This study was carried out between 2019 and 2020 in 12 Portuguese residential care homes (RCH). The following cluster eligibility criteria were considered: (1) RCHs that receive youths aged between 12 and 25 years old, located in the center region of Portugal were included; (2) RCHs specialized in mental and behavioral disorders and/or substance abuse problems were excluded, because they adopt different and specific intervention models. Within the selected RCHs, caregivers who were directly involved in the delivery of services to youth on a regular basis were invited to collaborate.

A total of 127 professional caregivers accepted to participate. Randomization took place at the cluster level, after baseline. Six RCHs were allocated to the treatment group (66 caregivers; 52%), and six RCHs were allocated to the control group (61 subjects; 48%). From the six RCHs allocated to the treatment group, four RCHs were mixed and two received only females, accommodating from 15 to 45 children and youth. These RCHs had between 10 and 21 professionals with different roles. The six RCHs of the control group accommodated from 15 to 40 children and youth, four were mixed and two were gender specific, one for females, and the other one for males. These RCHs had between 14 and 23 professionals with different roles. All RCHs included in this study were 24/7 open group homes (e.g., youth attend local public schools, they are integrated in community sports, and visit their families), they are located in urban and rural areas on the center of Portugal mainland, and receive mostly nationwide children and youth referred by the child protection services. In Portugal, RCHs have the main goal of time-limited protection of youth at-risk, aiming to ensure their safety, well-being, education, and healthy development. Most placements are due to maltreatment (e.g., neglect and psychological, physical and sexual abuse), and the remaining are due to abandonment by caregivers or the lack of family support (ISS, 2022). All RCHs have a technical (e.g., technical director, psychologist, social worker), educational (e.g., educators, direct care staff), and support (e.g., cook, cleaning staff) teams, being supervised by the public Welfare Services. Staff from educational and support teams often do not have an academic degree or pre-service training, and might work in rotating shifts. In accordance with a recent assessment of the quality of care in Portuguese RCHs, most of the RCHs revealed not to use evidence-based practices or interventions (Rodrigues & Barbosa-Ducharne, 2017).

Participants were mostly female (89%), and were aged between 22 and 62 years old, with a mean age of 43.99 (SD = 10.96). The majority of participants were married (69%), 23% were single and 7.9% were divorced. Participants had been working within RYC settings for less than a month to 39 years (M = 11.95, SD = 8.99), having a technical (29.4%; e.g., management, psychologist, social worker), educative (63.5%; e.g., educational assistant) or support (7.1%; e.g., cleaning staff, cooker) function. Half of them (52%) reported they were working in shifts. Concerning educational level, 44.1% had a higher education degree, 19.7% reported having completed high school, and 36.2% some level of elementary or middle school education. No significant differences between groups were found in sociodemographic features (cf. Table 1).

**Table 1:** Sociodemographic features by group

	Treatment group		Control group		*t*	*p*	Cohen’s *d*
	*M*	*SD*	*M*	*SD*			
Age	42.92	10.90	45.15	11.00	− 1.143	0.255	0.204
Years of work in RCH	10.77	7.89	13.25	9.98	− 1.536	0.127	0.276
	*N*	%	*N*	%	χ^2^	*p*	Cramer’s *V*
Gender							
Male	5	7.6	9	14.8	1.665	0.197	0.115
Female	61	92.4	52	85.2			
Marital status							
Single	19	28.8	10	16.7	2.926	0.232	0.152
Married	43	65.2	44	73.3			
Divorced	4	6.1	6	10.0			
Education degree							
Elementary/middle school	24	36.4	22	36.1	5.710	0.058	0.212
High school	8	12.1	17	27.9			
Higher education degree	34	51.5	22	36.1			
Profession							
Technical director	6	9.1	2	3.3	6.104	0.729	0.220
Psychologist	8	12.1	6	10.0			
Social worker	5	9.1	3	5.0			
Social educator	6	9.1	6	10.0			
Educational assistant	34	51.5	36	60.0			
Socio-educational animator	2	3.0	1	1.7			
Teacher	1	1.5	0	0			
Cleaning staff	1	1.5	4	6.7			
Cooker	3	4.5	2	3.3			
Staff functions							
Technical	22	33.3	15	25.0	1.152	0.562	0.096
Educative	40	60.6	40	66.7			
Support	4	6.1	5	8.3			
Shifts							
Yes	36	54.5	30	49.2	0.366	0.545	0.054
No	30	45.5	31	50.8			

Elementary and middle school education correspond to 4–9 years of school; High school are 12 years of school; Higher education degree are Bachelor or Master degrees

#### Sample Size

Effective sample size was determined for usual factorial (2 groups) repeated measures (4 assessments) between factors, using G*Power, version 3.1.9.7, considering alpha = 0.05, a medium effect size (Cohen’s f = 0.36) to obtain at least 80% power, assuming a 0.80 correlation coefficient within repeated measures. Under these assumptions, a total of 54 caregivers should be enrolled, 27 in each experimental condition.

### Intervention

The CMT-Care Homes is a manualized program developed for professional caregivers working in RYC. Main goals are to cultivate a compassionate-self and foster a caregiving mentality in RYC. It is strongly based on the Compassion Focused Therapy theoretical framework (e.g., affect regulation systems, flows, and fears of compassion) and Compassion Mind Training practices (e.g., compassionate imagery, soothing rhythm breathing, compassionate letter) (Gilbert, 2020) applied to the RYC needs and practices. The CMT-Care Homes is made of twelve 2-h and half structured sessions, offered once a week, during approximately three months. Sessions took place at the workplace in a group format, ranging from 6 to 12 participants.

All sessions present the same structure (check-in, exploration, and check-out). Program contents are organized across three modules: (1) Our mind according to a compassion-based approach (to provide insight into the evolved and socially shaped mind and the affect regulation systems; composed of 6 sessions); (2) Compassionate mind training (understanding and cultivating the attributes and competencies of the three flows of compassion, and addressing its fears; composed of 5 sessions); and (3) a Final session (revising key information/practices, and its application into the RCH; composed of 1 session). Contents are explored through psychoeducation and experiential practices followed by group opportunities to debate and share experiences. Considering that the transfer of learnings from training to everyday life constitutes a recurrent problem in RYC (Liu & Smith, 2011), throughout the program, participants are invited to reflect on how session’s learnings can be transferred into: 1) their own daily routine (e.g., self-regulation, self-care, balance between personal and professional life); 2) their relationship with children and youth (e.g., understanding their behavior and using adequate strategies to help them to regulate their emotions); 3) RCHs’ practices and routines (e.g., team work, communication). A compassionate weekly challenge, including between sessions training of formal meditation practices and compassionate learnings, is also given to encourage the transference of CMT-Care Homes’ learnings to caregivers’ daily routine and work tasks.

### Measures

#### The Professional Quality of Life Scale, Version 5 (ProQOL-5; Stamm, 2010; Portuguese Version by Carvalho, 2011)

ProQOL is a 30-item self-report scale designed to measure the positive and negative effects of working in stressful environments. ProQOL is composed of three subscales: Compassion Satisfaction (CS), Burnout (BO) and Secondary Traumatic Stress (STS). Participants are instructed to indicate how frequently each item was experienced in the workplace, during the previous 30 days, using a 5-point scale (1 = never, to 5 = very often). The original version reported internal consistency values of 0.88 for CS, 0.75 for BO, and 0.81 for STS (Stamm, 2010). The Portuguese version also showed good internal consistency (CS α = 0.86, BO α = 0.71, STS α = 0.83) (Carvalho, 2011). In this study, Cronbach’s alphas were 0.81 for CS, 0.64 for BO and 0.67 for STS.

#### Depression, Anxiety and Stress Scales (DASS-21; Lovibond & Lovibond, 1995 ; Portuguese Version by Pais-Ribeiro et al., 2004)

DASS-21 is a 21-item self-report scale designed to assess symptoms of depression, anxiety, and stress. Participants are asked to rate how much each statement applied to them during the previous week, using a 4-point scale (0 = not apply at all to me, to 3 = applied to me most of the time). In the original version, the DASS-21 subscales presented high internal consistency: Depression α = 0.91, Anxiety α = 0.84, and Stress α = 0.90 (Lovibond & Lovibond, 1995). The Portuguese version showed good internal consistency (Depression α = 0.85, Anxiety α = . 74, Stress α = 0.81) and good convergent and discriminant validity (Pais-Ribeiro et al., 2004). In this study, Cronbach’s alphas were 0.87 for depression, 0.86 or anxiety and 0.87 for stress.

#### Self-Compassion Scale (SCS; Neff, 2003; Portuguese Version by Castilho et al., 2015)

SCS is a 26 self-reported scale designed to assess self-compassion. Participants are instructed to answer the items regarding “how I typically act towards myself in difficult times”, using a 5-point scale (1 = almost never, to 5 = almost always). In the original version, the scale has a total score (α = 0.92) and six subscales (Self-Kindness, Self-Judgement, Common Humanity, Isolation, Mindfulness, and Over-Identification), with alpha values ranging from 0.75 for Mindfulness to 0.81 for Over-Identification (Neff, 2003). In the current study, we used the two-factor model found in the Portuguese version: Self-Compassionate attitude (comprising the positive subscales: Self-Kindness, Common Humanity, Mindfulness) and Self-Critical attitude (comprising the negative subscales: Self-Judgement, Isolation and Over-Identification), with alpha coefficients of 0.91 and 0.89, respectively (Costa et al., 2015). In the current study, Self-Critical attitude was used as a covariate (α = 0.88).

#### Pandemic Related Questions

Considering that Covid-19 pandemic co-occurred with 3 and 6-month follow-up assessments, a brief questionnaire was developed to address the level of anxiety that the pandemic triggered on caregivers. It was assessed through one item question (“Please indicate the level of anxiety that the current situation of COVID-19 causes you”), ranging from 0 “nothing” to 10 “extremely”. For the treatment group, the questionnaire also addressed the level of usefulness of the CMT-Care Homes to deal with the pandemic (“Please indicate to what extent CMT-Care Homes is useful to deal with the current situation of COVID-19, e.g., fear, anxiety, change in routines and habits, social isolation, uncertainty regarding the future”) and with children and youth during lockdown measures (“Please indicate how useful the CMT-Care Homes is to deal with children and young people in residential care during the current COVID-19 pandemic”), using the same scale.

### Procedures

Written informed consent was sought at the cluster (i.e., boards of each RCH) and at the individual level (i.e., caregivers), before randomization. Participants were informed of the goals and procedures, and were asked to voluntarily participate, with no incentives offered for participation. Anonymity was guaranteed, with the use of respondent-specific codes, which were also used to link the data from one timepoint to the other. Caregivers were assessed through self-report measures at baseline, post-treatment, 3 and 6-month follow-up. Considering that the Covid-19 outbreak started during the current CRT, at 3 and 6-month follow-up caregivers were also asked to answer to a questionnaire about the level of anxiety concerning pandemic and usefulness of the program in that context. Due to the pandemic situation, data were collected in person by a researcher assistant (when possible) or were sent to each RCH to be filled out individually. After the baseline assessment, a computer-generated randomization was conducted at the cluster level, following a completely randomized design by the third author of this paper. Each RCH (i.e., cluster) was randomly assigned to treatment or control group (i.e., no training in compassion or any other group interventions). The CMT-Care Homes program was delivered in accordance with the handbook, in a face-to-face format, weekly (2.5-h session) in each RCH, to a group of 6–12 participants, over approximately 3 months, from October 2019 to February 2020. All sessions were delivered by the first author, who is a clinical psychologist trained in cognitive-behavioral interventions and compassionate approaches.

### Data Analysis

Data was analyzed with IBM SPSS Statistics v25. Prior to analysis, data were screened for missing data, outliers, and multivariate analysis of covariance (MANCOVA) assumptions (Tabachnick & Fidell, 2013). Missing data were examined by incidence and distribution, both by subject and per item. Five participants presenting more than 20% of missing values in an outcome variable were removed (Peng et al., 2006). Little's (1988) MCAR tests revealed that data in some outcome variables were not missing completely at random (*p* < 0.05). Considering that deletion of cases would lead to a substantial loss of subjects, missing values of participants with less than 20% of missing data in one outcome variable were dealt via linear interpolation imputation method (Meyers et al., 2006).

Baseline differences between the two groups were examined for demographics and for outcome variables, via independent samples t-tests and chi-square statistics. The effect sizes were calculated, using Cohen’s d, with 0.15 indicating a small effect, 0.36 a medium effect and 0.65 a large effect (Lovakov & Agadullina, 2021); and Cramer’s V, with 0.10 indicating a small effect, 0.30 a medium effect, and 0.50 a large effect (Cohen, 1988).

Although confirming normal univariate distribution by coefficients of skewness and kurtosis (SK <|3| and Ku <|10|; Kline, 2005), with skewness values ranging from − 1.126 to 2.057 and kurtosis values ranging from − 0.564 to 4.963, data did not reveal a multivariate normal distribution (assessed via Mardia’s test; Korkmaz et al., 2014). Violations of normality can, however, be disregarded considering the absence of multivariate outliers (investigated via Mahalanobis distance; Tabachnick & Fidell, 2013). The homogeneity of variance–covariance matrices was ensured (assessed via Box’ M test, p > 0.001) (Field, 2018). Multicollinearity was absent, since correlations between outcome variables were < 0.90 (Tabachnick & Fidell, 2013).

To investigate intervention effects on the multiple outcomes, a two-factor (i.e., between subjects—groups—and within subjects—time) mixed MANCOVA was conducted. In accordance with former research, baseline levels of self-critical attitude were controlled due to individual differences in self-criticism on the response to compassion-based interventions (e.g., chronically self-critical individuals have more difficulties in accessing self-reassuring imagery; Duarte et al., 2015; Gilbert et al., 2004; Matos et al., 2022a), and its role as a major vulnerability factor for several mental disorders (Werner et al., 2019). Education degree was entered as a co-variate in the analysis, considering its possible influence on stress levels (Del Valle et al., 2007; Santos et al., 2022a).

For MANCOVA multivariate test, the Pillai’s criterion was used, as it is considered most robust when assumptions are not fully met (Field, 2018). Sphericity was analyzed via Mauchly’s W. When this assumption was not verified, the Greenhouse–Geisser epsilon were checked and when ε $>$0.75 Huynh–Feldt criterion was used in univariate tests. Effect sizes for the time effects and time × group effects were calculated using partial eta squares (${\eta }_{p}^{2}$), with ${\eta }_{p}^{2}$ = 0.01 referring to a small effect size, 0.06 to a medium effect size and 0.14 to a large effect size (Tabachnick & Fidell, 2013). To understand group differences, Cohen’s d was computed for long-term changes.

Pearson correlations were computed between anxiety related with pandemic and outcome measures for follow-up assessments.

## Results

### Recruitment and Retention

**Fig. 1:**
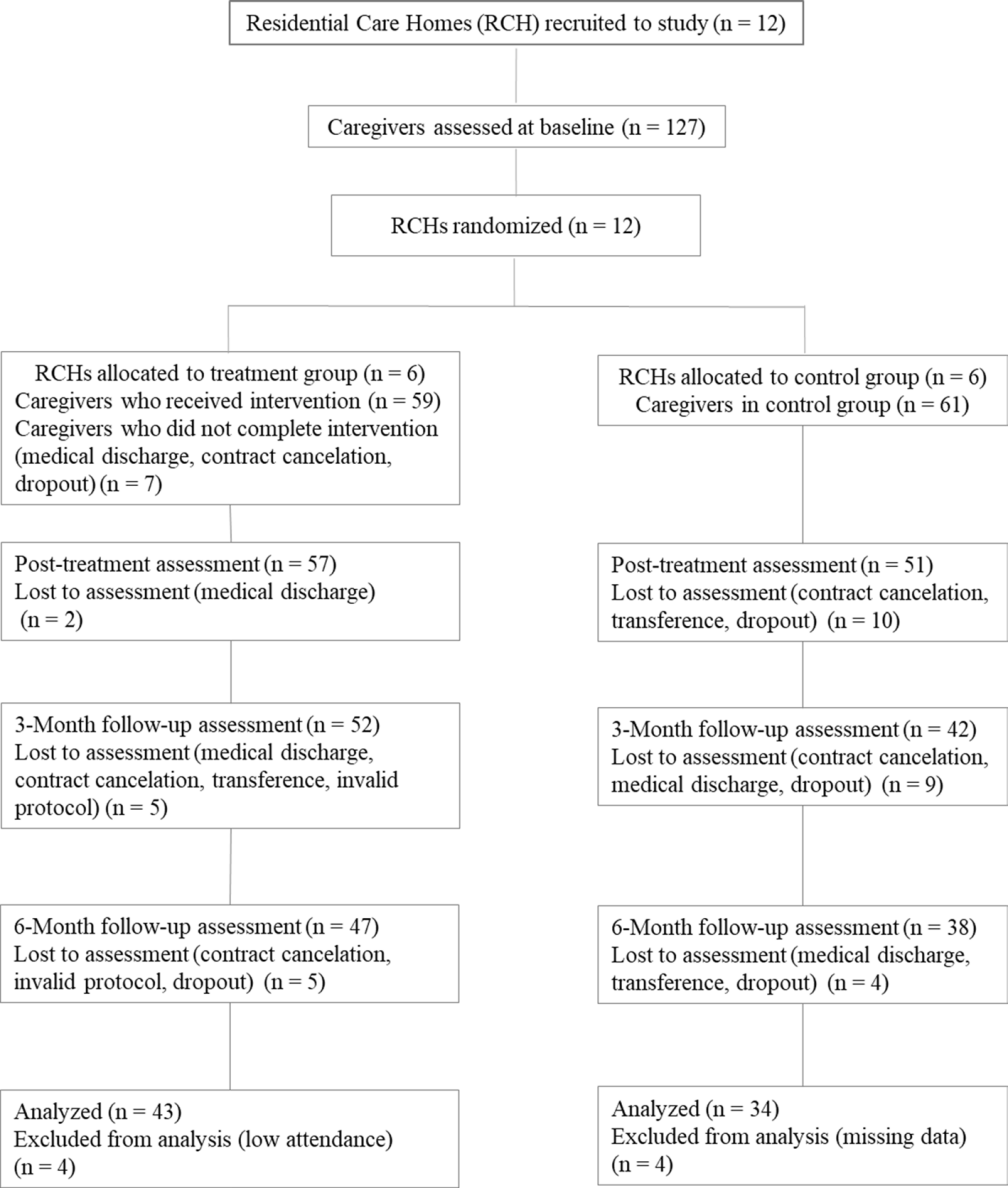
Flowchart of caregivers’ participation

All caregivers from the 12 RCHs accepted to participate and completed the baseline assessment (N = 127) (cf., Fig. 1). RCHs were randomly distributed to the treatment (6 RCH; N = 66 caregivers) and control (6 RCH; N = 61 caregivers) groups. Of the initial 66 participants allocated to the treatment group, seven (10.61%) did not complete the program: two withdrew due to cancellation of the job contract, three due to prolonged medical discharge, and two dropped out from intervention. Fifty-nine (89.39%) participants completed the program and 57 the posttreatment assessments (86.36%; two caregivers were in medical discharge at time of assessment). Five participants (7.58%) were lost to assessment at 3-month follow-up: two of them due to job contract cancelation, one was transferred to another social response, one was at medical discharge, and one protocol was invalid. Another five participants were lost to assessment at 6-month follow-up (7.58%): two of them due to contract cancelation, two had invalid protocols, and one dropped out. Caregivers within this condition attended 5 to 12 sessions (*M* = 9.52; *SD* = 1.99). The main reasons for not completing the whole program were working in shifts/day off, vacation, brief medical discharge or urgent professional diligences. Four caregivers (6.78%) who attended less than 60% of the sessions were excluded from the analyses.

Of the 61 caregivers allocated to the control group, 51 (83.61%) completed the posttreatment assessment, one left the study due to cancellation of the job contract, one was transferred to another social response, and eight dropped out the study. Nine participants were lost to assessment at 3-month follow-up (14.75%): four of them due to contract cancelation, four due to medical discharge, and one dropped out. At 6-month follow-up four participants were lost to assessment (6.56%): one due to medical discharge, one was transferred to another social response, and two dropped out. Four participants (6.56%) were excluded from analyses due to missing data. In total, 77 caregivers (89.6% females), aged between 22 and 62 years old, were included in the analysis, at the experimental (*N* = 43) or control (*N* = 34) groups. At 3-month follow-up, 72.1% of the participants at CMT-Care Homes continued to practice exercises and 92.9% still applied the learnings related with the program. At 6-month follow-up percentages were 61.9% and 78.6%, respectively.

### Baseline Differences

No significant differences were found between treatment and control group at the onset of the study for demographics (cf., Table 1) and outcome measures (all *p* > 0.05) (cf., Table 2).

**Table 2:** Baseline differences on the outcome measures

Measures	Treatment group		Control group		*t*	*p*	Cohen’s *d*
	*M*	*SD*	*M*	*SD*			
ProQOL-5							
Compassion satisfaction	37.81	4.69	36.41	4.51	1.618	0.108	0.304
Burnout	24.35	4.30	24.71	4.25	− 0.452	0.652	0.085
Secondary traumatic stress	25.94	4.47	25.47	4.80	0.539	0.591	0.104
DASS-21							
Depression	3.16	3.68	3.25	3.75	− 0.121	0.904	0.023
Anxiety	3.45	4.12	2.39	3.48	1.483	0.141	0.278
Stress	6.07	4.08	5.87	3.91	0.262	0.794	0.049

### Two-Factor Mixed MANCOVA

Multivariate tests, with self-critical attitude and education degree as covariates, demonstrated a significant Time × Group interaction effect (Pillais’ trace = 0.150, F = 1.890, *p* = 0.014) corresponding to a small effect size (${\eta }_{p}^{2}$ = 0.050). Univariate tests for Time × Group interaction, with the same covariates, indicated that when compared with the control group, the treatment group had significantly lower scores in burnout, depression and anxiety (cf., Table 3). These differences corresponded to small to moderate effect sizes. No significant differences were found between groups for stress, compassion satisfaction, and secondary traumatic stress.

**Table 3:** Mean scores and standard deviations for both groups at baseline, posttreatment, 3 and 6-months follow-up, and univariate test

	Treatment group				Control group				ICC (CI)	Time	Time × Group
	T1	T2	T3	T4	T1	T2	T3	T4			
	M (SD)	M (SD)	M (SD)	M (SD)	M (SD)	M (SD)	M (SD)	M (SD)			
ProQOL											
CS	38.00 (4.99)	39.08 (4.26)	38.97 (4.54)	38.79 (4.07)	37.09 (3.44)	37.41 (4.08)	37.04 (5.14)	36.61 (4.16)	0.87 (0.82–0.91)	*F* = 0.860; *p* = 0.463; ɳ^2^_p_ = 0.012	*F* = 0.861; *p* = 0.462; ${\eta }_{p}^{2}$ = 0.012
BO	24.05 (4.59)	23.48 (3.81)	22.60 (3.79)	22.77 (3.86)	23.96 (3.65)	23.38 (3.07)	24.38 (3.50)	23.47 (3.86)	0.85 (0.79–0.90)	*F* = 0.636; *p* = 0.129; ɳ^2^_p_ = 0.009	*F* = 3.393; *p* = 0.021; ${\eta }_{p}^{2}$ = 0.044
STS	25.42 (4.33)	24.86 (4.54)	24.23 (4.36)	24.19 (3.92)	24.62 (3.70)	23.74 (4.06)	24.18 (3.70)	24.21 (3.25)	0.84 (0.77–0.89)	*F* = 0.356; *p* = 0.769; ɳ^2^_p_ = 0.005	*F* = 1.230; *p* = 0.299; ${\eta }_{p}^{2}$ = 0.017
DASS											
DEP	2.28 (3.21)	1.19 (2.00)	1.71 (1.89)	1.07 (1.47)	2.62 (2.73)	2.03 (2.19)	2.54 (2.40)	2.91 (2.97)	0.67 (0.53–0.77)	*F* = 0.527; *p* = 0.664; ɳ^2^_p_ = 0.007	*F* = 2.954; *p* = 0.033; ${\eta }_{p}^{2}$ = 0.039
ANX	2.64 (3.37)	1.35 (1.77)	1.56 (2.37)	1.02 (1.81)	1.32 (1.34)	1.41 (2.12)	1.43 (2.01)	2.00 (2.41)	0.61 (0.45–0.74)	*F* = 0.120; *p* = 0.949; ɳ^2^_p_ = 0.001	*F* = 5.837; *p* = 0.001; ${\eta }_{p}^{2}$ = 0.074
SS	4.88 (3.62)	3.98 (3.35)	4.15 (3.74)	3.63 (3.02)	4.94 (2.91)	4.32 (2.69)	4.99 (3.29)	4.70 (3.83)	0.70 (0.57–0.79)	*F* = 0.044; *p* = 0.988; ɳ^2^_p_ = 0.093	*F* = 1.086; *p* = 0.356; ${\eta }_{p}^{2}$ = 0.015

*CS* compassion satisfaction, *BO* Burnout, *STS* secondary traumatic stress, *DEP* depression, *ANX* anxiety, *SS* stress. T1 = preintervention; T2 = postintervention; T3 = 3-month follow-up; T4 = 6-month follow-up; *M* mean, *SD* standard deviation, *ICC* intraclass correlation coefficient, *CI* confident interval (95%); ${\eta }_{p}^{2}$ = partial eta square

When examining means, standard deviations, and corresponding effect sizes (Cohen's d for each group), results showed that caregivers from the treatment group gradually reduced their burnout levels from preintervention to 6-month follow-up (Cohen's d = 0.30). In turn, for the control group, burnout levels were kept stable from preintervention to 6-month follow-up (Cohen's d = 0.13).

In what concerns depression, caregivers from the treatment group progressively improved from preintervention to 6-month follow-up (Cohen's d = 0.49), while the control group did not change across time (Cohen's d = 0.10). Regarding anxiety symptoms, the treatment group gradually decreased anxiety levels from preintervention to 6-month follow-up (Cohen's d = 0.60). In turn, the control group showed a tendency for increasing anxiety from preintervention to 6-month follow-up (Cohen's d = 0.35). These differences corresponded to small to moderate effect sizes.

### The Impact of Covid-19 Pandemic

Anxiety related with the Covid-19 outbreak was correlated with outcome variables measured at follow-up assessments, showing significant positive, but weak, associations (all r < 0.40). Exceptions were for stress and compassion satisfaction. At 3-month follow-up, corresponding to the onset of the pandemic and lockdowns, stress and anxiety related to the pandemic achieved a significant and moderate positive correlation (r = 0.406), but this association was not significant anymore at 6-month follow-up, when lockdown measures were relaxed. Compassion satisfaction and anxiety related with the pandemic did not correlate at 3-month follow-up, but showed a significant and weak negative correlation at 6-month follow-up (r = -0.293).

Groups significantly differed at the level of anxiety associated with the pandemic at 3-month follow-up (*t* (73) = -2.295; *p* = 0.025; treatment group *M* = 5.74, *SD* = 2.72 and control group *M* = 7.06, *SD* = 2.14), with controls showing higher levels. Groups did not significantly differ at 6-month follow-up (*t* (72) = -1.366; *p* = 0.176; treatment group *M* = 5.93, *SD* = 2.47 and control group *M* = 6.67, *SD* = 2.10).

Although CMT-Care Homes was not designed to deal with a pandemic, caregivers recognized the program usefulness when dealing with the contingencies associated with the Covid-19 outbreak (3-month follow-up *M* = 6.81, *SD* = 2.28; 6-month follow-up *M* = 6.90, SD = 2.46) and with children and youth during lockdowns (3-month follow-up *M* = 7.62, *SD* = 1.83; 6-month follow-up *M* = 7.48, *SD* = 2.29).

## Discussion

The current study intended to expand the preliminary evidence on the CMT-Care Homes (Santos et al., 2022b) and test its effectiveness on caregivers’ professional quality of life and mental health outcomes, within a cluster randomized trial in a real-world setting. The CMT-Care Homes aims to cultivate a compassionate mindset on caregivers, not only through promoting compassion towards others, which sets the base for any helping profession, but also through promoting self-compassion and the openness to receive compassion from others. This would facilitate feelings of safeness with others and help to improve emotion regulation (Preckel et al., 2018; Vachon, 2016). Alongside the cultivation of compassion (Santos et al., 2022b, 2023b), the CMT-Care Homes was expected to reduce suffering and psychological distress as well. To the best of our knowledge, this is the first compassion-based program delivered to caregivers working in RYC settings.

At baseline, groups did not yield significant differences on demographic and outcome measures. These results may indicate that randomization was effective, allowing for reliable conclusions on the CMT-Care Homes’ effects.

A multivariate analysis of covariance (MANCOVA) was carried out in order to test for intervention effects on professional quality of life and mental health outcomes. When controlling for education degree and self-critical attitude at baseline, MANCOVA revealed statistically significant Time x Group interaction effects of the CMT-Care Homes on burnout, depression, and anxiety, with small to medium effect sizes. Improvements in the treatment group were observed at follow-ups. These findings are in line with previous research, highlighting the beneficial effect of compassionate-based interventions on psychological distress and mental health symptoms (Irons & Heriot-Maitland, 2020; Kirby et al., 2017; Matos et al., 2017). Similar findings were also found using compassion-based interventions in organizations (Andersson et al., 2022), and specifically in caring-focused environments, with caregivers of patients with intellectual disabilities (Sansó et al., 2017) and teachers in school settings (Matos et al., 2022a).

Our findings indicated improvements from baseline to 6-months follow-up for burnout, anxiety, and depression. Research involving compassion-based interventions also revealed the maintenance of changes (Irons & Heriot-Maitland, 2020; Matos et al., 2022a) or continued improvements in depression symptoms at follow-ups (Ferrari et al., 2019). Other programs designed for the RYC setting, using other theoretical frameworks, did not show significant improvements on burnout (Donald, 2015) and mental health (Vallejos et al., 2016). Hence, both the compassion training and its theoretical framework seem a promising approach to counteract burnout, anxiety, and depression on caregivers, helping to improve their functioning and the quality of the care they provide.

Previous research suggested that the maintenance and improvements in changes may be related with the practice and the transference of learned techniques and strategies into the daily routine (Maratos et al., 2019). In fact, in the present study, more than 70% of participants having received the CMT-Care Homes reported to have kept practice three months after the program completion and more than 90% reported they were still applying the learnings related with the program during 3-month follow-up assessments. At 6-month follow-up, percentages decreased, but were still over 50%. This might have contributed to the observed improvements in mental health symptoms, even when facing new challenges linked to the pandemic context.

In contrast, and for the control group, burnout and depression levels did not change from baseline to 6-month follow-up, and anxiety symptoms seemed to have gradually increased. This might suggest that, in face of usual demands of this particular care setting, plus the additional challenges of the pandemic, when no training or support is offered, caregivers’ mental health might tend to deteriorate over time, which might have negative implications both for their own quality of life, as for the quality of the care they provide (Sinclair et al., 2021). Also, the level of anxiety related with the pandemic reported on the first follow-up, which co-occurred with the first lockdown at the onset of the COVID 19 pandemic in Portugal, was significantly higher for the control than for the treatment group. Accordingly, participants in the CMT-Care Homes recognized the usefulness of the program in dealing both with the contingencies associated with the pandemic and with children and youth during the lockdowns. Although it has not been designed to deal with the Covid-19, a training of this nature seems to be helpful to cope with stressful events like a pandemic. This is in line with research conducted during the pandemic outbreak, which demonstrated the protective role of compassion on mental health, by buffering the harmful effects of the Covid-19 (Matos et al., 2022b).

Compassion satisfaction, STS, and stress did not reveal significant differences between groups across time. Compassion satisfaction refers to the pleasure derived from being able to provide care to others (Stamm, 2010). It is important to recognize that other organizational factors that are beyond the scope of this program (e.g., work overload, low payment) may have influenced these outcomes. In addition, compassion satisfaction changes have not been found in other studies with caregivers from other care settings (Delaney, 2018; Matos et al., 2022a; Pfaff et al., 2017; Potter et al., 2013). Similarly, a resiliency program including self-care strategies and mindfulness to deal with compassion fatigue in a health care setting did not achieve changes on compassion satisfaction or STS (Pfaff et al., 2017). STS commonly occurs in professionals who deal with traumatized clients, developing their own symptoms of traumatic stress and similar reactions as posttraumatic stress disorder (PTSD, e.g., re-experiencing, avoidance and hyperarousal; Bride et al., 2007). Hence, 12-group sessions may have not been sufficient or even adequate to treat a clinical condition such as STS. It is also important to emphasize that most participants did not report high levels of STS at baseline. Thus, this finding might be attributed to the sample’s apparent floor effect, as it occurred in a former randomized controlled trial of acceptance and commitment therapy for social workers (Brinkborg et al., 2011), which did not find significant effects for professionals with low levels of stress at baseline.

In what concerns stress, stress levels were found to be moderately correlated with perceived threat of COVID-19 reported at the 3-month follow-up. Considering that the pandemic onset and its additional challenges (Carvalho et al., 2022; Ravens-Sieberer et al., 2020; Whitt-Woosley et al., 2022) co-occurred with follow-up assessments, this might have somehow influenced the results. This finding is aligned with research suggesting that the perceived threat of COVID-19 was associated with higher scores in stress (Matos et al., 2022b).

This CRT provides evidence about a new program to support the mental health and the quality of life of professionals working within RYC. The CMT-Care Homes covers the research gaps reported on a recent systematic review (Santos et al., 2023a) and is aligned with international recommendations to protect the RYC staff well-being (Whittaker et al., 2016). It also extends the research regarding compassion-based interventions in helping settings to RYC, showing that a compassion training may have a buffering effect over caregiver’s mental health concerns. Findings also suggested that, when no training is offered, caregivers tend to deteriorate their mental health across time. Considering the personal and organizational costs of caring, organizations should prioritize staff training and support in order to protect caregivers’ well-being and to prevent staff turnover, which is a significant threat to the implementation of new models and evidence-based practices (Steinlin et al., 2017). Additionally, and in order to overcome the continuous changes in staffing as one of the major challenges to maintain effectiveness of trainings over time (Ogden & Fixsen, 2015), it seems essential that RCH psychologists can be trained to deliver the program to future staff members. To do so, training budgets, often viewed as dispensable when organizational cuts are made, must be protected (Hofmeyer et al., 2020).

Some limitations should be kept in mind when considering the findings of the current study. First, despite using standardized measures, the exclusive reliance on self-report instruments might encompass associated bias. Since psychophysiological measures (e.g., hair or salivary cortisol, heart rate variability) have been used to assess psychophysiological correlates of compassion, emotion regulation, and stress (Schmid et al., 2020; Sousa et al., 2021), future research should resort to such measures as a way to strengthen self-report findings. Second, the sample size prevented resorting to more sophisticated statistics to analyze longitudinal data (e.g., latent growth curve models). Other studies have also reported difficulties in collecting longitudinal data in these settings due to the rotativity of staff linked with turnover (Schmid et al., 2020; Turner, 2017; Vallejos et al., 2016). Nevertheless, future research should replicate this study with larger samples and in another time, in order to investigate the CMT-Care Homes effectiveness outside of pandemic related constraints.

To conclude, findings highlight the utility and relevance of compassion-focused interventions in RYC settings, showing the potential benefits of the CMT-Care Homes in helping caregivers to develop socio-emotional competencies for caring for their own mental health and professional quality of life, while taking care of vulnerable youth.

## Data Availability

The datasets generated and analysed during the current study are available from the corresponding author upon request. The corresponding author takes responsibility for the integrity of the data and the accuracy of the data analysis.

## Code Availability

The custom code generated during the current study are available from the corresponding author upon request.
